# Ovotesticular disorder of sex development in a 46 XY adolescent: a rare case report with review of the literature

**DOI:** 10.1186/s12905-023-02698-1

**Published:** 2023-10-24

**Authors:** Koui Bbs, Abouna Ad, Toukilnan Djiwa, Traore B, Kouyate M, Kouame Ke, Aman Na

**Affiliations:** 1 Department of Pathological Anatomy, Teaching Hospital of Treichville, Treichville, Côte d’Ivoire; 2 Department of Pathological Anatomy, Teaching Hospital of Lomé, University of Lomé, Lomé, BP 1515, Togo; 3 Department of Pathological Anatomy, Teaching Hospital of Bouaké, Bouaké, Côte d’Ivoire

**Keywords:** Ovotestis, Adolescent, Histology, Karyotype, Disorders of Sex Development (DSD)

## Abstract

**Introduction:**

: Ovotestis is a rare cause of sexual ambiguity characterized by the presence in a patient of both testicular and ovarian tissue, leading to the development of both male and female structures. We report a case of ovotestis diagnosed in an adolescent, with a review of the literature.

**Case Report:**

A 15-year-old patient presented with a right scrotal swelling associated with gynecomastia. Histology showed a juxtaposition of ovarian stroma with ovarian follicle and seminiferous tubules. Karyotype revealed a male subject (XY). We have therefore retained the diagnosis of ovotesticular disorders of sex development.

**Conclusion:**

Ovotestis is a rare finding, heterogeneous in its genetic etiology and clinical presentation. While many patients are diagnosed during infancy or childhood, we presented a case diagnosed in a 15-year-old adolescent.

**Supplementary Information:**

The online version contains supplementary material available at 10.1186/s12905-023-02698-1.

## Introduction

The term ‘Disorders Of Sex Development’ (DSD) is now proposed to define congenital conditions in which a dysharmony between chromosomal, gonadal and anatomical sex exists [1]. Ovotestis is a rare cause of sexual ambiguity characterized by the presence in a patient of both testicular and ovarian tissue, leading to the development of male and female structures [1–3]. The incidence and constitutive karyotype of patients with ovotestis are reported to vary geographically [4, 5]. Ovotestis accounts for 3–10% of all sex ambiguities [2]. Its incidence in South Africa is estimated at 4% [6]. The prevalence of ovotestis is estimated to be less than 1:20,000, and approximately 500 individuals have been reported to date [7]. Sex ambiguity is often discovered at birth. Karyotypes vary; 46,XX, 46,XY and several mosaic and aneuploid forms [1].

We report a case of ovotestis diagnosed in a 15-year-old adolescent, describe the clinical presentation and diagnostic features.

## Case report

A 15-year-old patient, with no previous pathological history, raised as a boy, consulted the urology department of the University Hospital of Treichville for a right scrotal swelling, evolving for more than 5 years. The patient began full and normal male puberty at the age of 13.

The clinical examination revealed a patient in good general condition, measuring 1.56 m and weighing 47 kg, corresponding to a body mass index of 19.31 kg/m². The patient had Tanner stage 2 pubic hair, with a penis measuring 4 cm at rest, without hypospadias or cryptorchidism.

**Fig. 1:**
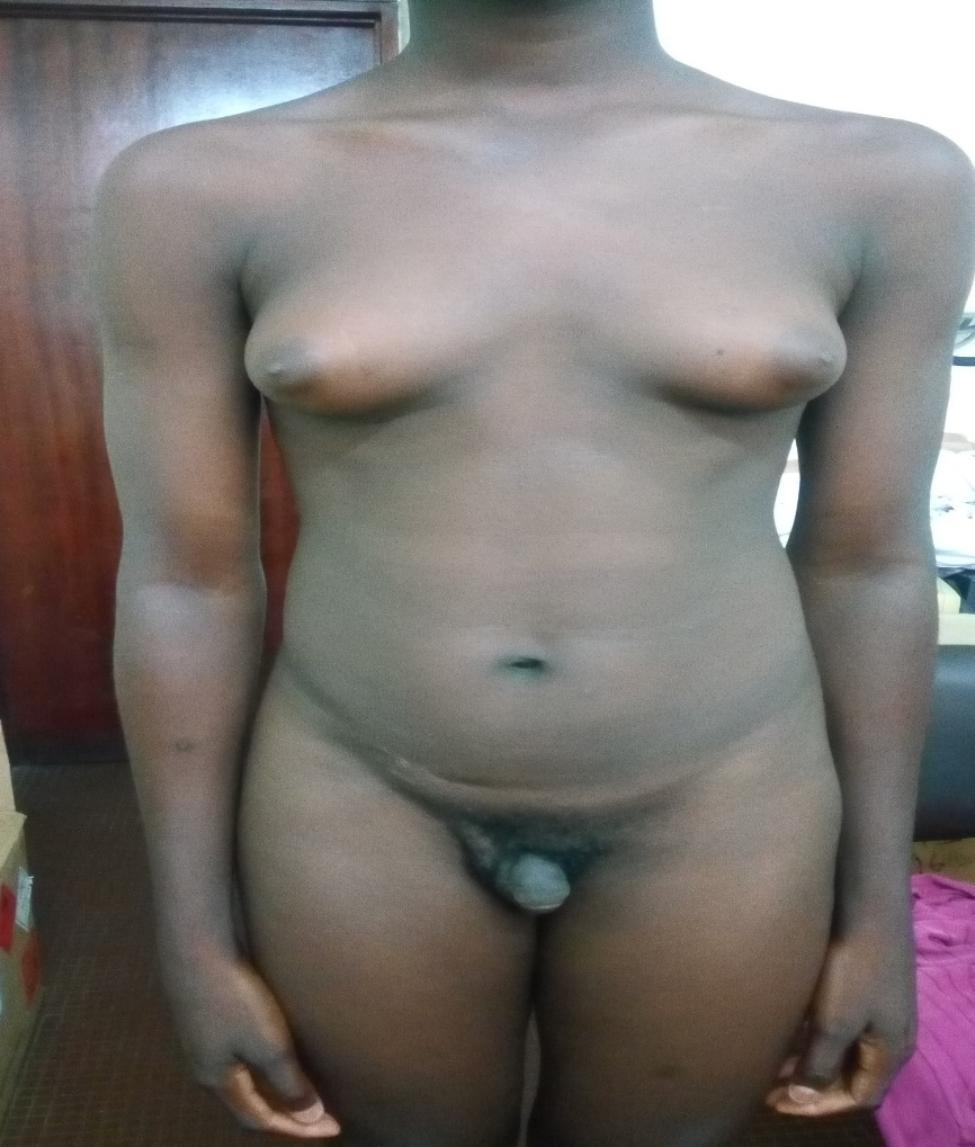
Male subject with bilateral gynecomastia

A painless, mobile and firm right scrotal mass was palpated. The left testicle was present but atrophic. He had a female morphotype, with marked gynecomastia (Fig. 1). The lymph nodes were free.

On pelvic and scrotal ultrasound, a right testicular solid tumor with a long axis of 6 cm was found, with absence of female genitalia. No uterus or adnexa was seen. The contralateral testis was without abnormality.

The biological workup consisted of TESTOSTERONE (0.1 ng/ml), ESTRADIOL (20 ng/ml), ALPHA-FETO-PROTEIN (5 ng/ml) and β HCG (< 01ui /l); all of which came back normal.

A right orchiectomy was performed and the specimen sent to the pathology laboratory.

**Fig. 2:**
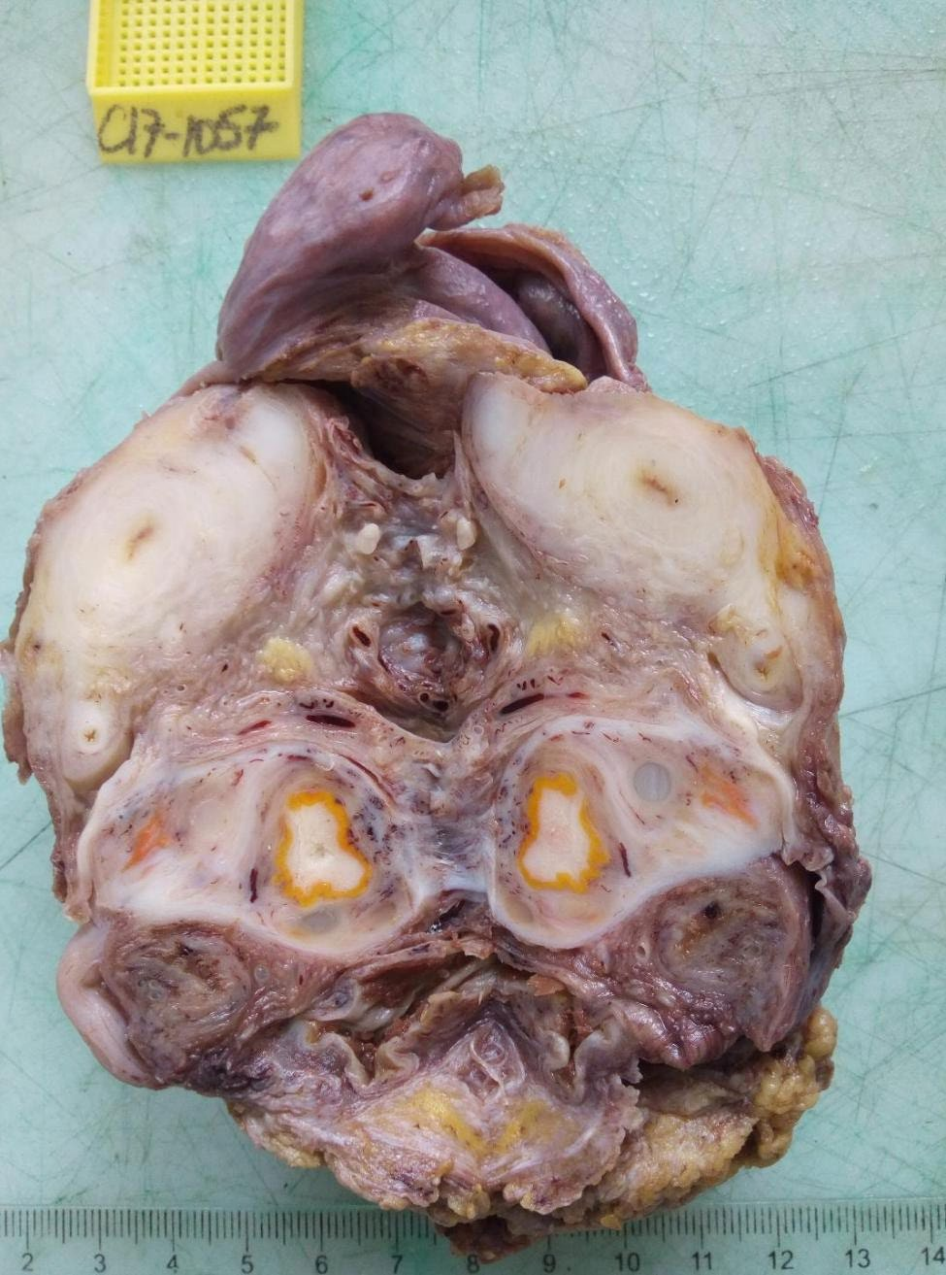
Whitish multinodular areas and whitish territories surrounded by a yellowish border

Macroscopically, it was a lumpy mass. On section, there were several whitish nodules surrounded by a yellowish border (Fig. 2).

**Fig. 3:**
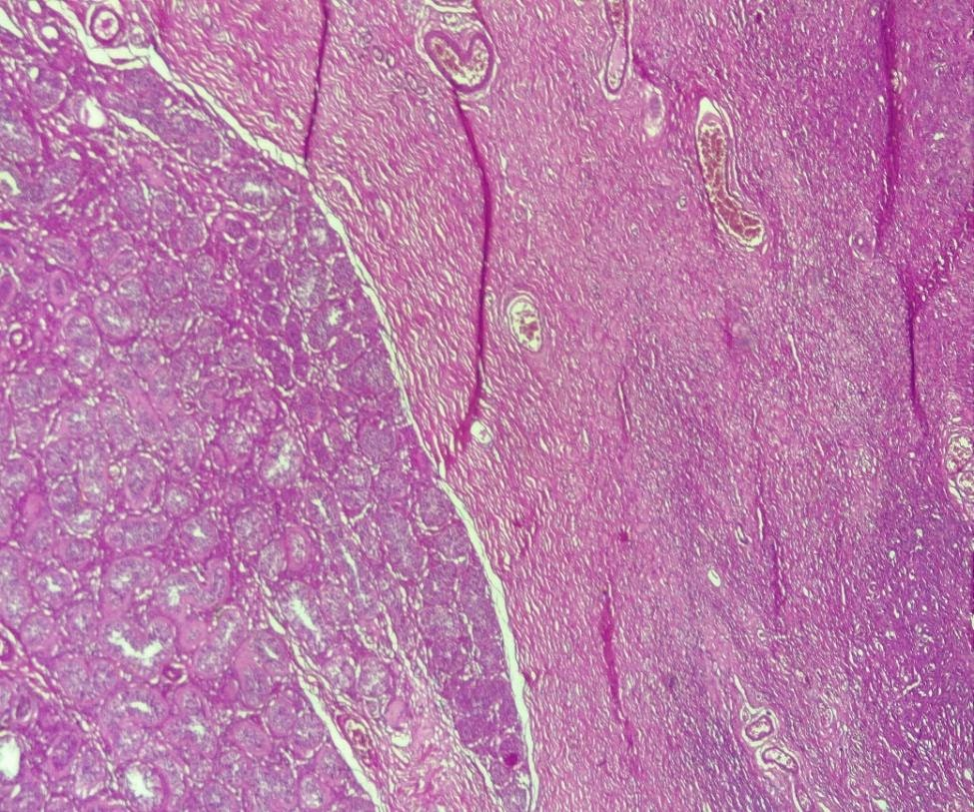
Juxtaposition of ovarian stroma and seminiferous tubules (HESx100)

**Fig. 4:**
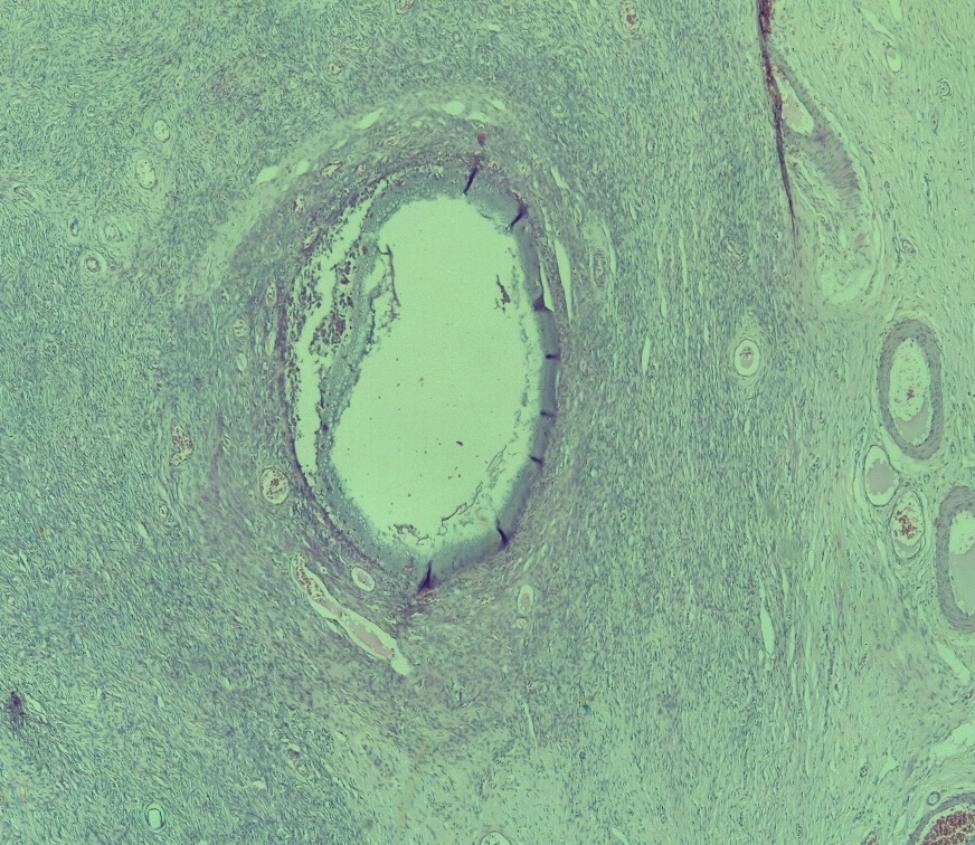
Ovarian tissue with a follicular cyst and an ovarian follicle (HESX100)

**Fig. 5:**
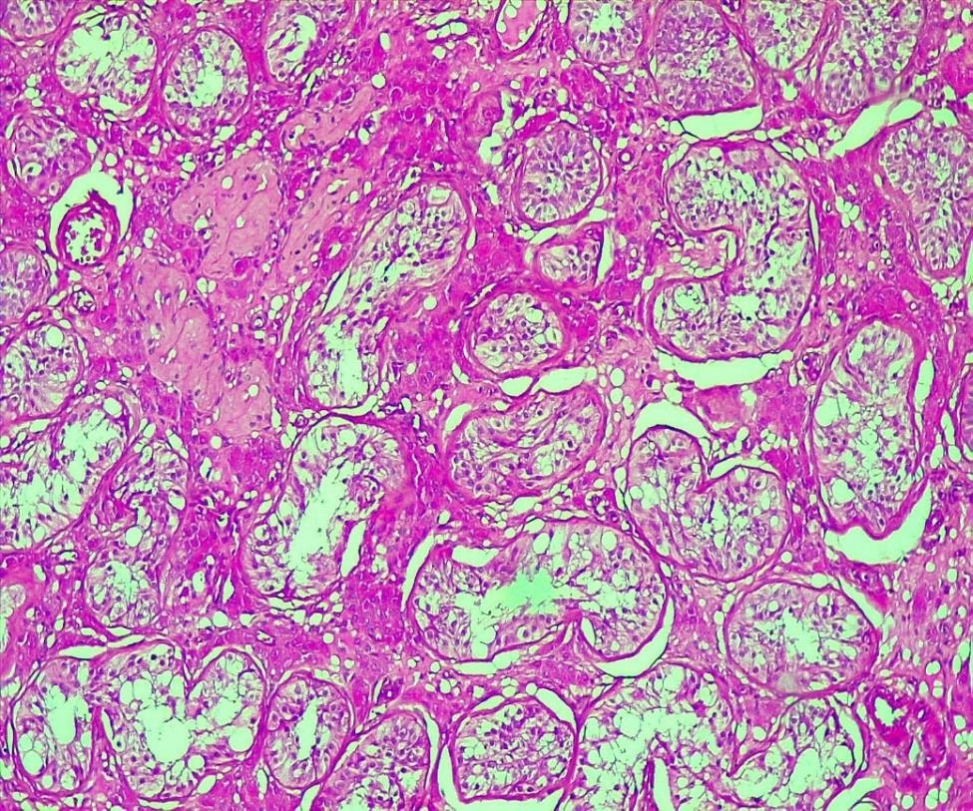
Testicular parenchyma seen at high magnification (HESX100)

The resected specimen was fixed with 10% formaldehyde, followed by conventional dehydration, kerosene embedding, sectioning and hematoxylin and eosin (HE) staining. On histology, there was a juxtaposition of ovarian stroma with ovarian follicle and seminiferous tubules (Figs. 3 and 4, and 5). In view of this aspect we retained the diagnosis of ovotestis. The karyotype was determined to be male (46, XY), confirming the diagnosis of ovotesticular disorders of sex development. An initial interview was held with the parents in the presence of a psychologist to explain their child’s pathology, before the child was informed during a second interview in the presence of the parents and the psychologist.

Hormonal therapy based on 80 mg testosterone undecanoate capsules at a dose of once a day and mastectomy were proposed, but the patient was lost to follow-up.

## Discussion

Sexual ambiguities are congenital conditions with atypical chromosomal, gonadal or anatomical sex development [8]. They occur in one in 4500 births and ovotestis accounts for 3–10% of all sex ambiguities [2]. Defined as congenital conditions in which development of chromosomal, gonadal, or anatomic sex is atypical, differences or disorders of sex development (DSDs) comprise many discrete diagnoses ranging from those associated with few phenotypic differences between affected and unaffected individuals to those where questions arise regarding gender of rearing, gonadal tumor risk, genital surgery, and fertility [9]. Controversies exist in numerous areas including how DSDs are conceptualized, how to refer to the set of conditions and those affected by them, and aspects of clinical management that extend from social media to legislative bodies, courts of law, medicine, clinical practice, and scholarly research in psychology and sociology [9, 10]. In addition to these aspects, this review covers biological and social influences on psychosocial development and adjustment, the psychosocial and psychosexual adaptation of people born with DSDs, and roles for clinical psychologists in the clinical management of DSDs [11].

Approximately 400 cases of ovotestis have been reported to date [8]. Although ovotestis is rare [12–14], it is thought to be more prevalent in black South Africans [8], particularly ovotesticular sex ambiguity 46,XX. Most cases are sporadic with few documented cases of familial recurrence [8]. It is often diagnosed at birth [1]. We report a case of ovotestis, diagnosed in a 15 year old adolescent of female morphotype with bilateral gynecomastia, who consulted for a right scrotal swelling. Clinical and ultrasound examinations revealed a right scrotal mass with an atrophic testicle, and a normal biological work-up. A large series of 20 Brazilian patients with ovotestis was diagnosed at a mean age of 11 years. Clinical features included bilateral gynecomastia and cyclic hematuria [15]. Clinical features often depend on the underlying karyotype. The most common karyotype in ovotestis is 46, XX [4, 15]. Our patient had a 46, XY karyotype. Amolo et al. found a predominance of 46, XY karyotype in 46.2% [8]. In the Indonesian series of Juniarto et al., 63.3% of the patients had 46, XY karyotype [16]. Individuals with 46, XY karyotype have the highest risk of developing a malignant gonadal tumor, due to the presence of a non-functional testicular component in the ovotestis, which requires removal of the non-functional gonads [17–20]. Emphasis is placed on a multidisciplinary approach in order to maximize the potential of these individuals to become normal socially functioning adults [20]. Hormone replacement therapy as well as mastectomy were proposed in our case, but the patient was lost to follow-up.

## Conclusion

Ovotestis is a rare finding, heterogeneous in its genetic etiology and clinical presentation, its diagnosis is based on comprehensive clinical information, coupled with pelvic ultrasound and histopathology results, confirmed by karyotype. While many patients are diagnosed in infancy or childhood, we reported a case diagnosed in a 15-year-old adolescent with a 46, XY karyotype. This was diagnosed late due to normal male phenotypic development over time, with the need for long-term follow-up to assess hormonal status and sexual function. Because of the high risk of developing gonadal cancer in these patients, emphasis is placed on long-term multidisciplinary management, including psychologists, endocrinologists, gynaecologists, paediatricians and even plastic surgeons.

### Electronic supplementary material

Below is the link to the electronic supplementary material.

Supplementary Material 1

## Data Availability

All data supporting the conclusions of this article are included in the manuscript.

## Abbreviations

DSD: Disorders/ Differences of Sex Development
